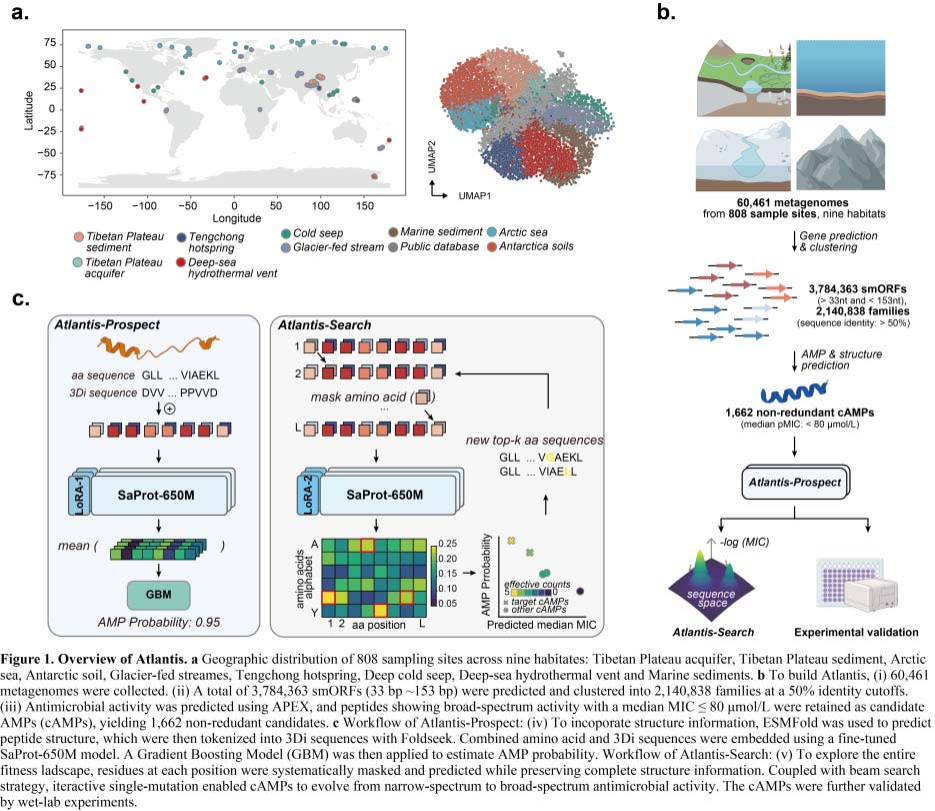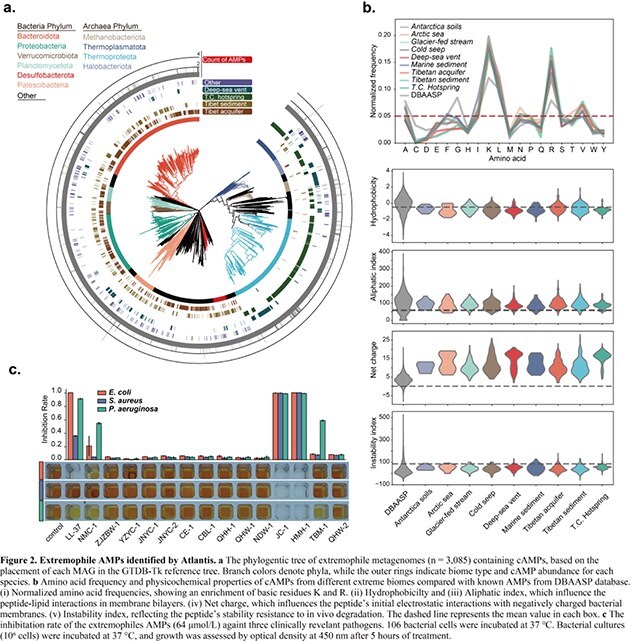# Discovery and optimization of antimicrobial peptides from extreme environments on global scale

**DOI:** 10.1093/bib/bbaf631.027

**Published:** 2025-12-12

**Authors:** Zixin Kang, Haohong Zhang, Kang Ning

**Affiliations:** MOE Key Laboratory of Molecular Biophysics of the Ministry of Education, Hubei Key Laboratory of Bioinformatics and Molecular-imaging, Center of AI Biology, Department of Bioinformatics and Systems Biology, College of Life Science and Technology, Huazhong University of Science and Technology, Wuhan 430074, Hubei, China; MOE Key Laboratory of Molecular Biophysics of the Ministry of Education, Hubei Key Laboratory of Bioinformatics and Molecular-imaging, Center of AI Biology, Department of Bioinformatics and Systems Biology, College of Life Science and Technology, Huazhong University of Science and Technology, Wuhan 430074, Hubei, China; MOE Key Laboratory of Molecular Biophysics of the Ministry of Education, Hubei Key Laboratory of Bioinformatics and Molecular-imaging, Center of AI Biology, Department of Bioinformatics and Systems Biology, College of Life Science and Technology, Huazhong University of Science and Technology, Wuhan 430074, Hubei, China

## Abstract

**Background:**

Antimicrobial peptides (AMPs) inhibit microbial growth through membrane disruption or interference with intracellular processes, offering promising solutions to antimicrobial resistance (AMR). While AMPs have been extensively identified and verified from animal proteomes, reference microbial genomes and host environments, those from extreme habitats remain largely unexplored. Microbes in such extreme niches with low-level of nutrients evolve unique membrane modification and specialized metabolic pathway, representing a hidden reservoir for novel AMP discovery.

**Methods:**

We developed Atlantis, a language model-driven framework for AMP detection and optimization from global extremophile (Fig. 1). It combined two modules: Atlantis-Prospect, which predict antimicrobial potency based on both sequence and structure information, and Atlantis-Search, which conducts iterative single-mutations with structural constraints to broaden peptides’ antimicrobial activity from narrow to broad spectrum. The pretrained structure-aware language model was fine-tuned on small proteins from extremophile to capture biome-specific evolutionary information.

**Results:**

By mining 60,461 extremophile metagenomes from nine discrete habitats across multiple geographical scales, we identified 1662 non-redundant peptides with potential antimicrobial activity from 85 bacterial and archaeal phyla, none of which match existing databases. We synthesized fourteen peptides, and two of them showed stronger antimicrobial activity then commercial AMP, LL-37, in vitro against *Escherichia coli*, *Pseudomonas aeruginosa* and *Staphylococcus aureus* (Fig. 2).